# Surgical management of pyelo-ureteral junction syndrome in a resource-limited setting: case of Zinder National Hospital, Niger

**DOI:** 10.1186/s12893-019-0609-2

**Published:** 2019-10-23

**Authors:** Harissou Adamou, Ibrahim Amadou Magagi, Maazou Halidou, Hassane Diongolé, Mahamadou Doutchi, Oumarou Habou, Kabirou Ganiou, Amadou Soumana, Rachid Sani

**Affiliations:** 1 General Surgery and Surgical Specialties Department, National Hospital Zinder, Zinder, Niger; 2 Faculty of Health Sciences, University of Zinder, Zinder, Niger; 3 Urology Department, National Hospital of Zinder, Zinder, Niger; 4 Nephrology-dialysis Department, National Hospital of Zinder, Zinder, Niger; 5 Infectiology Department, National Hospital Zinder, Zinder, Niger; 6 Medical Imaging Department, Zinder National Hospital, Zinder, Niger; 7 Urology Department, National Lamordé Hospital, Faculty of Health Sciences, University of Niamey, Niamey, Niger; 8 Department of Surgery, National Hospital of Niamey, Faculty of Health Sciences, University of Niamey, Niamey, Niger

**Keywords:** Pyelo-ureteral junction, Open surgery, Pyeloplasty

## Abstract

**Background:**

Pyelo-ureteral junction syndrome (PUJS) is a frequent congenital malformation. We report the surgical management of PUJS by pyeloplasty according to Anderson-Hyne-Kuss’s procedure at the Zinder National Hospital..

**Methods:**

This was a retrospective study from January 2013 to December 2016 (4 years), including patients who have undergone surgery for PUJS.

**Results:**

Twelve (12) cases of PUJS had a surgery among which 66.7% were men with an average age of 32.5 ± 7.6 years. The clinical symptomatology was lumbar pain or renal colic in 92.3% of cases. This pain had evolved for more than 2 years for 58.3% of the cases. Ultrasound coupled with intravenous urography or CT-scan was performed to confirm the diagnosis of PUJS in 58.3 and 41.7% of cases. The average serum creatinine level at admission was 181.25 ± 67.3 μmol/L [Lab reference range: 53–97 μmol/L]. The Anderson-Hynes non dismembered pyeloplasty is used for all the patients. The release of a crossing lower pole vessel was performed in 25%, pyelolithotomy in 16.7%. The average surgery time was 118.3 ± 20.7 min. The average hospital length of stay was 10.8 ± 3 days. Immediate postoperative complications were recorded in 33.3% (*n* = 4). Postoperative outcomes were considered good by disappearance of clinical, biological and radiological signs.

**Conclusion:**

The Anderson-Hynes non dismembered pyeloplasty gives good results and provides a successful alternative in an environment where laparoscopy and robotic surgery are not developed.

## Background

Pyelo-ureteral junction syndrome (PUJS), also known as primary hydronephrosis, is a common cause of urinary malformation in the upper urinary tract [1–4]. It is characterized by a narrowing of the junction between the pelvis and the ureter [1, 2, 4–6]. PUJS may result from an intrinsic, functional, congenital abnormality of the ureteral wall or extrinsic compression more often by a lower polar vessel [2, 3, 6]. In developed countries, its diagnosis, which is increasingly occurring during the antenatal period, and neonatal management have improved its prognosis [1, 6, 7]. In resource-limited settings, the diagnosis is often carried out during clinical manifestations, sometimes in adulthood [2, 8, 9]. Since Anderson-Hynes’s first description in 1949 [10], open pyeloplasty has been the gold standard in the surgical treatment of PUJS with a success rate greater than 90% [2, 4, 5, 11]. Today, with the progress of minimally invasive surgery in developed countries, pyeloplasty by laparoscopic or robotic approaches is supplanting open surgery; in fact, the minimally invasive approaches produce the same success rates, with aesthetic advantages, low morbidity, and short convalescence [1, 3, 6, 12]. In sub-Saharan Africa, open surgery is still the most used [2, 8, 9, 11]. We hereby report the surgical management of PUJS by pyeloplasty according to Anderson-Hyne-Kuss’s procedure at the Zinder National Hospital.

## Methods

This was a retrospective study of the period from January 2013 to December 2016 at the Urology Department of Zinder National Hospital. This study included all adult patients, of at least 18 years of age, who have undergone pyeloplasty for pyelo-ureteral junction syndrome. Patients who did not have a surgery were not included. Unusable files of PUJS cases were excluded. Cases of non-functionning kidney requiring nephrectomy were excluded.

Zinder National Hospital (ZNH) is a tertiary level hospital, located somewhere about 900 km from Niamey, the capital city of Niger Republic. The bed capacity was 800. The Zinder region has an estimated population of 4,132,321 inhabitants in 2016 (Ministry of Health). This hospital also receives patients from neighboring regions.

Patient records, outpatient and operating room registers were used to collect the data. The classification of Valayer and Cendron was used to group patients based on the degree of pyelocalyceal dilatation [13]. Type I: localized dilation at the pelvis at the inferior convex border downwards; type II: dilation of the pelvis and calices, but rapid impregnation of the cavities and good thickness of the parenchyma; type III: large pyelocalicic dilation with a fuzzy and incomplete image within normal delays, very thinning of the cortex; type IV: silent kidney [13]. A cytobacteriological examination of the urine was done to all of the patients and the antibiotic treatment was instituted if necessary, before surgery.

**Fig. 1:**
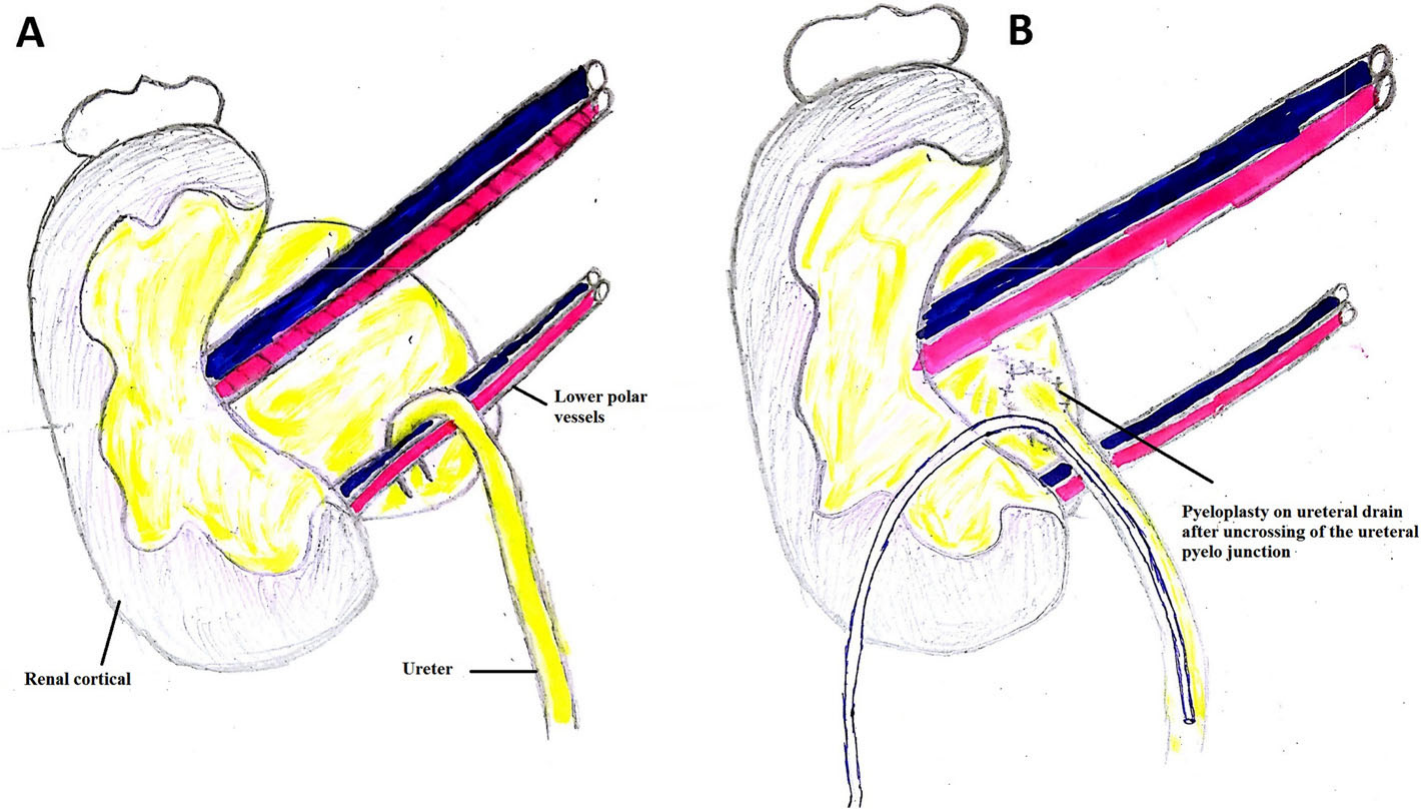
**a** Appearance of crossing by a lower polar vessel, **b** Postoperative appearance after uncrossing and pyeloplasty

A lumbar incision passing through the 12th rib allowed to approach the operative site. The surgical procedure consisted of resection of the narrowed pyelo-ureteric junction with pyeloplasty by separate points according to the Anderson-Hynes-Kuss procedure with placement of a ureteral drain [10, 14, 15]. When there was a lower compressive polar vessel, an uncrossing was performed before pyeloplasty (Fig. 1).

An aspirating Redon drain was left in the renal lodge and was removed after 48 h, if it did not bring back any fluid. Nephrectomy was necessary in case of parenchyma completely destroyed. In the case of bilateral localization, pyeloplasty was performed on the most symptomatic side, taking into account the clinical (pain) and radiological criteria (degree of dilation). The patient being taken for a second time for the treatment of contralateral side.

The following parameters were studied: age, sex, clinical and radiological signs, operative aspects (surgery duration, intraoperative findings and procedures), postoperative complications and follow-up. The follow-up of the patients who have undergone surgical treatment was done after 1 month, 3 months and 6 months with clinical exam, ultrasound, urography and biological control (renal function and cytobacteriological examination of the urine). The surgery was considered successful when there was a disappearance of the pain and a radiological improvement (absence of hydronephrosis, passage of contrast medium to urography). The patients’ consent to publish the results as well as local ethics approval was obtained.

## Results

During the study period, 12 cases of PUJS underwent surgery. Men accounted for 66.7% (*n* = 8) and women, 33.3% (*n* = 4), a sex ratio of 2. The average age was 32.7 ± 7.9 years (range: 20–46 years). Clinical symptomatology with low back pain (53.8%) or renal colic (38.5%) was the constant reason for consultation. This pain has been evolving for more than 12 months for 5 patients, more than 2 years for 5 patients, and more than 5 years for 2 patients. The physical examination showed a good general status for all patients.

**Fig. 2:**
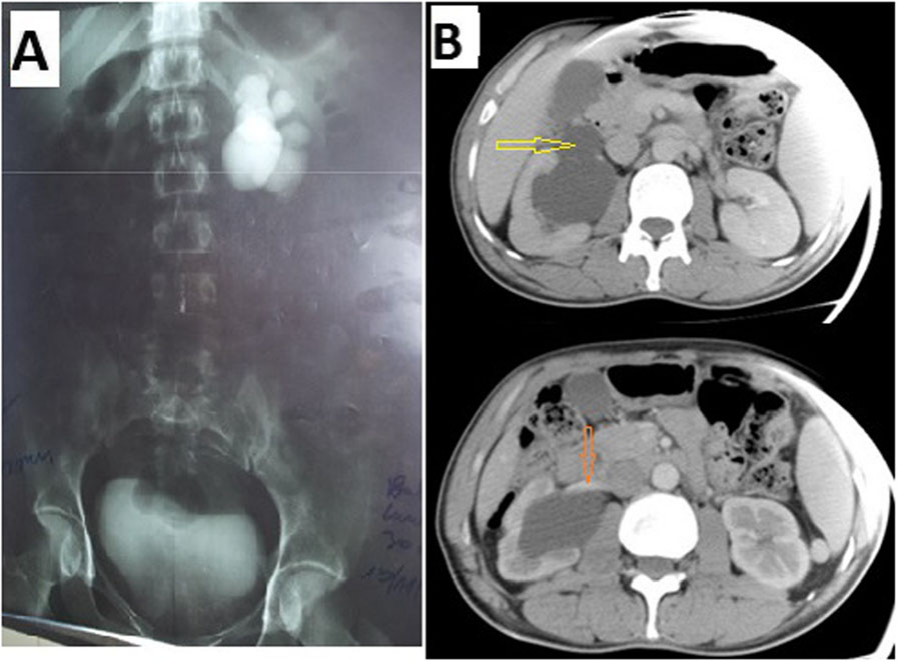
**a** Intravenous urography: Left pyelic dilation in ball with convex lower edge and non-opacified subpyretic ureter. **b** Injected abdominal CT scan in axial section showing pyelic dilatation (yellow arrow) compressed in front by a lower polar renal vein (orange arrow)

Palpation of the lumbar region revealed pain and lumbar contact with 25% (*n* = 3). Hydronephrosis was diagnosed in all patients by abdominal ultrasonography. Intravenous urography and CT-scan (Fig. 2) were performed to confirm the diagnosis of PUJS in 58.3% (*n* = 7) and 41.7% (*n* = 5), respectively. Pyelic lithiasis was found in 16.7% (*n* = 2).

The right side was reached in 58.3% (7 cases). Pyelocalyceal dilation (PCD) was type III in 58.3% (7 cases), type II in 41.7% (5 cases). The mean serum creatinine level at admission was 181.25 ± 67.3 μmol/L. Table 1 summarizes the different characteristics of the patients.

**Table 1:** Socio-epidemiological, clinical, paraclinical and perioperative characteristics of patients

N°	Age (year)	Sex	Clinical signs	Creat1 μmol/L)	Creat2 (μmol/L)	CBUE^a^	Localization	Type PCD^b^	OT^c^	Operative procedures	DUPD^d^	DRD^e^	Complications	LOS^f^
1	33	M	Lumbar pain	160	85	Sterile	Right	II	110	Pyeloplasty	3	10	Surgical site infection	10
2	46	M	Lumbar pain	220	75	Sterile	Right	III	100	Pyeloplasty	3	5	None	8
3	24	M	Renal colic	280	88	*Pseudomonas aeruginosa*	Left	III	120	Uncrossing + pyeloplasty	4	6	None	9
4	28	F	Lumbar pain	175	105	Sterile	Right	III	145	Pyeloplasty	4	25	Urinary leakage	11
5	36	M	Renal colic	300	90	*Klebsiella pneumoniae*	Bilateral	III	165	Right pyeloplasty	6	12	None	10
6	42	M	Lumbar pain	120	95	Sterile	Right	III	110	Pyeloplasty	5	14	Surgical site infection	18
7	30	F	Renal colic	210	85	Sterile	Left	II	100	Uncrossing + pyeloplasty	3	4	None	12
8	38	M	Renal colic	150	74	Sterile	Right	II	90	pyeloplasty	4	5	None	10
9	37	F	Lumbar pain	230	98	*Proteus mirabillis*	Bilateral	III	120	Right pyeloplasty	3	4	None	6
10	23	F	Lumbar pain	90	65	Sterile	Right	III	120	Pyeloplasty	6	15	Surgical site infection	15
11	35	M	Lumbar pain	130	80	Sterile	Right	II	110	Uncrossing + pyeloplasty	2	3	None	12
12	20	M	Renal colic	110	85	Sterile	Left	II	130	Pyeloplasty	3	5	None	9

^a^Cytobacteriological Urine Exam, ^b^pyelocalyceal dilation, ^c^Operating time in minutes, ^d^Duration of the uretero-pyelic drain (days), ^e^Duration of retroperitoneal drainage (days), ^f^Length of hospital stay. Creat1: serum creatinine level at admission, Creat2: serum creatinine level at 6 months

**Fig. 3:**
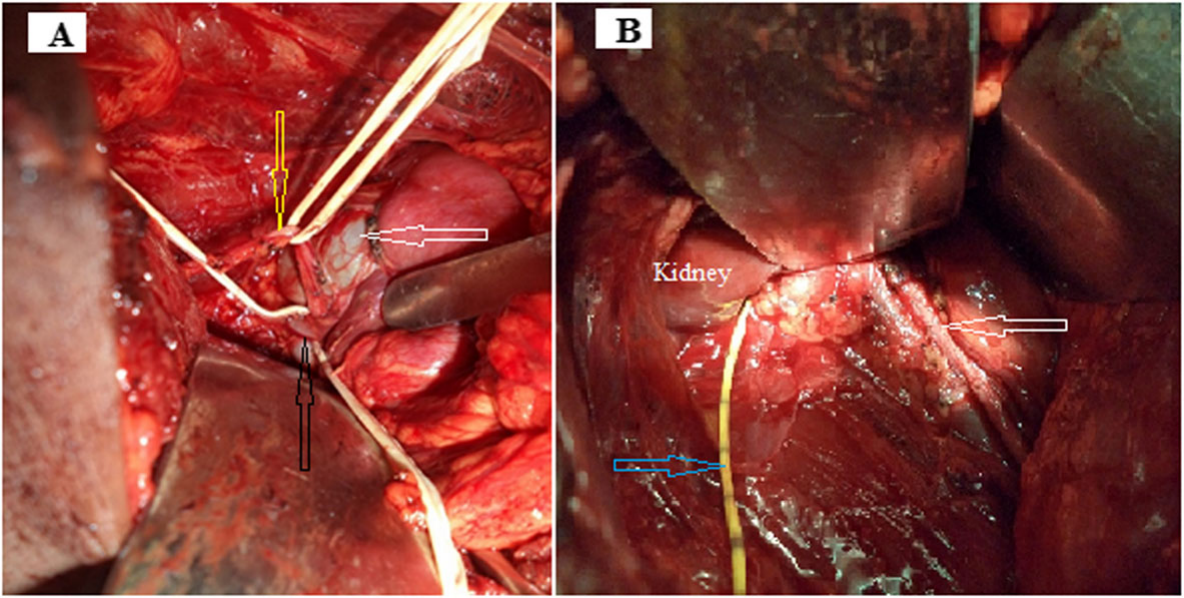
**a** Ureter (yellow arrow) and inferior polar vein (black arrow) compressing the pyelo-ureteral junction with pyelic dilatation (white arrow), **b** trans-nephro-pyelo-ureteral drain (blue arrow), ureter after descending and pyeloplasty (white arrow)

The Anderson-Hynes open pyeloplasty technique was performed for all patients. The crossing of a lower polar vessel was involved in 25% (3 cases) and the treatment consisted of an uncrossing followed by a pyeloplasty with intubation by a trans-nephro-pyelo-ureteral drain (Fig. 3). A pyelolithotomy was performed on to two patients. Drainage of the lumbar lodge was systematic for all of the patients. The average duration of the procedure was 118.3 ± 20.7 min, (range: 90 and 165 min). In both cases where PUJS was bilateral, only the symptomatic side had surgery.

The lumbar lining drain was removed within a median time of 5.5 days (range: 3 and 25 days) and the uretero-pyelic drain within a mean of 3.8 ± 1.3 days (Table 1). The average length of hospital stay was 10.8 ± 3 days. Postoperative complications were recorded in 33.3% (*n* = 4): 3 cases of surgical site infections (SSI) and one case of late urinary leakage. However, 2 cases of lower back pain were reported in the follow-up. The intravenous urography performed in all patients was normal with a good passage of the contrast medium. Renal function gradually normalized; after a follow-up of 6 months, all patients had a good renal function, with a mean serum creatinine level at 87.3 ± 12.6 μmol/L. (Table 2).

**Table 2:** Distribution of patients according to the follow-up of the renal function and the degree of pyelocalyceal dilation (Valayer-Cendron)

Creat (μmol/L)	Before surgery		3 months after surgery		6 months after surgery	
	GVC II	GVC III	GVC II	GVC III	GVC II	GVC III
80–120	1	2	4	2	5	7
[120–160]	3	–	1	3	–	–
[160–300]	–	6	–	2	–	–

*GVC* Grade of Valayer-Cendron classification

## Discussion

The main limitation of this study is its retrospective nature. The files review is possible because of the effective availability of all records found in the documentation department. The long-term follow-up is still missing to sufficiently appreciate the results of this open surgery. Once the surgical decision was made, the only alternative remains the Anderson-Hines procedure for all of the patients with or without a surgical history, in our context. However, the short-term results allow us to discuss with the literature data.

The syndrome of the pyelo-ureteral junction (PUJS) is a condition characterized by a lack of evacuation of urine from the pelvis to the ureter. It is an abnormality of intrinsic or extrinsic origin whose prevalence varies between 2 and 29 cases per 10,000 to 20,000 live births [1, 6, 16, 17]. Among adults, PUJS can develop idiopathically or in response to various traumatic attacks and the prevalence is estimated at 1 case per 1500 adult persons [1, 6, 16].

Studies carried out in Niger Republic are not available, this could have helped compare the prevalence of PUJS to the data found in literature. Our small series cannot be explained by a low prevalence in the Zinder region, but rather by under-reporting of cases for lack of diagnostic means and our study included only adult patients. The male predominance (66.7%) found in our series is described by several studies [2–4, 8, 9, 11, 17, 18]. The average age of 32.7 years in our study is consistent with the data found in literature. Most of the authors found an average age ranges between 26 and 36 years [2, 4, 8, 9, 11]. Moalic et al. [12] reported an average age of 44.8 years.

Lumbar pain is the main clinical sign leading patients to consult [2, 4–9, 11, 12, 18]. Clinical signs depend on age at diagnosis; PUJS is a disease that can occur in the perinatal period as well as in adulthood. In developing countries such as Niger Republic, diagnosis is often made in adulthood during a painful clinical manifestation [2, 8, 9, 18]. The paraclinical diagnosis is essentially made by the pair ultrasound-intravenous urography or ultrasound-CT-scan [2–4, 6]. Antenatal or neonatal PUJS is poorly reported in a context of limited resources [2, 8, 9, 18]. In our series, lithiasis was found in 15.4%. In the series of Tembely et al. [2], a lithiasic complication was associated with 17%. Diao et al. [11] reported 10% pyelolithotomy in their series. Studies are concordant on the involvement of PUJS in the development of urolithiasis and other metabolic abnormalities [2, 11, 16].

In our study, all of the patients had undergone an open surgery. Pyeloplasty was performed using the Anderson-Hynes-Kuss technique with trans-nephro-pyelo-ureteral drainage intubating the anastomosis and drainage from the renal lodge. Some authors recommend transpyelo-ureteral drainage to reduce hemorrhagic risks [11]. We had no haemorrhagic complications in our series. Kirakoya et al. [9] used three pyeloplasty techniques: Anderson-Hynes-Kuss pyeloplasty (37%), Bennassayag pyeloplasty (40%), and Culp tubular flap (2.9%). Kpatcha et al. [8] performed 24% Y-V plasty, and 68% resection-anastomosis according to Anderson-Hynes.

Anderson-Hynes open pyeloplasty used to be the gold standard in the surgical treatment of PUJS with therapeutic success that exceeded 90% [1–9, 11–19]. This technique has the advantage of performing a physiological pyeloplasty and also makes it possible to perform associated gestures such as uncrossing or ablation of a calculus [9, 11, 14]. This classic pyeloplasty is the procedure which is mostly used in many sub-Saharan African countries [2, 8, 9, 11, 18]. Indeed, this technique allows a good exposure of the renal compartment and therefore the achievement of easy sutures, but it has the disadvantage of causing postoperative pain, unsightly scars and incisional hernias [11, 14]. In our study, after 6 months of follow-up, renal function, cytobacteriological examination of the urine and urography were normal. For Elbaset et al. [20], pyeloplasty has a high rate of functional success even in patients with severe hydronephrosis and impaired renal function. These authors state that after 12 monts of follow-up, renal function has been improved and remained stable.

Even today, Anderson-Hynes-Kuss pyeloplasty enjoys some degree of praise in recurrent PUJS treatments or when the laparoscopic approach is difficult [7, 14, 21]. The advent of minimally invasive techniques (laparoscopy and endopyelotomy) is supplanting open surgery in the treatment of PUJS. Indeed, these techniques offer the same results in terms of therapeutic success with a significant aesthetic advantage, less postoperative pain and short hospitalization [1, 5–7]. Studies, which have been published since 2013 on the treatment of PUJS by using several techniques (pyeloplasty, endopyelotomy, laparoscopic pyeloplasty, robotic pyeloplasty and micro-laparoscopic pyeloplasty) confirm that laparoscopic and robotic surgery represents the current gold standard in the treatment of PUJS [1, 6, 22]. However, the growing trend of using robotic surgery was reported [1, 6]. These minimally invasive techniques, nevertheless, require a specialized technical platform and experienced surgeons. This remains a challenge for sub-Saharan Africa [2, 8, 9, 11, 18].

Due to the diversity of minimally invasive techniques in the treatment of PUJS, the surgical option must be based on several factors, such as success, morbidity of the technique, surgeon experience, cost and choice of patients [7]. For Brunet et al. [19], the quality of the pyelo-ureteral sutures determines the final result, regardless of the approach being used (open or laparoscopic surgery). The principles of anastomosis of Kuss, Anderson and Hynes must be respected [19]. This technique has the advantage of combining excellent exposure on pyelo-ureteral junction and performing a tight anastomosis [14, 15].

The presence of an inferior polar vessel leading to an uncrossing before pyeloplasty was found in 23% of cases in our study. We systematically performed a lower polar vessel uncrossing, as did Bentani et al. [4], because the obstruction of the pyelo-ureteral junction was evident (Figs. 1, 2 and 3). The number of PUJS cases recorded with a lower vessel as an obstacle to the evacuation of urine from the pelvis to the ureter varies from one series to another [1, 3, 4, 9, 11]. For Bentani et al. [4], a lower polar pedicle was found and uncrossed in 50% of cases. Kirakoya et al. [9] performed 2 inferior polar vessel decays in 4 cases recorded in their series. This uncrossing is also proposed laparoscopically by several authors. However it is advisable to remain cautious in the exploration of an inferior polar vessel, because the gesture can be perilous [3, 21].

In this study, the average time of surgery was 118.5 ± 19.8 min and the average length of stay at the hospital was 10.8 days. The data in our series are consistent with those found in the PUJS Open Surgery literature [2, 8, 9, 11, 14]. On the other hand, this operating time is classically higher in laparoscopic or robotic surgery, but with the advantage of reduced hospital stay and morbidity [1, 3–7, 12, 16]. Bentani et al. [4] reported an average response time of 175.1 min and a hospital length of stay of 3.46 days [4]. In the context of pyeloplasty, postoperative urinary leakage may be observed, as was the case in this study. In these cases of urinary leakage, we recommend keeping the retroperitoneal (peripyelic) drain until the end of the leak.

## Conclusion

The diagnosis of the pyelo-ureteral junction syndrome is often late in our context. Anderson-Hynes-Kuss’s procedure for PUJS treatment gives good results and remains the gold standard in an environment where laparoscopy and robotic surgery are not developed. However, it is clear that minimally invasive techniques are replacing open surgery in the management of PUJS; this requires a strengthening of the technical platform for the diagnosis and management of PUJS in Niger.

## Data Availability

Please contact corresponding author for data requests.

## Abbreviations

CBUE: Cytobacteriological Urine Exam
CT-scan: Computerized tomography
DRD: Duration of retroperitoneal drainage (days)
DUPD: Duration of the uretero-pyelic drain (days)
LOS: Length of hospital stay
OT: Operating time
PCD: Pyelocalyceal dilation
PUJS: Pyelo-ureteral junction syndrome
SSI: Surgical site infection
ZNH: Zinder National Hospital
